# Maternal and neonatal outcomes associated with multiple repeat cesarean deliveries: A registry-based study from Qatar

**DOI:** 10.5339/qmj.2024.3

**Published:** 2024-01-22

**Authors:** Shameena Ajmal, Thomas Farrell, Fathima Minisha, Sawsan Al Obaidly, Mai AlQubaisi, Najat Khenyab, Najah Ali, Husam Salama, Abdul Rouf Pallivalappil, Nader Al Dewik, Hilal Al Rifai

**Affiliations:** Department of Obstetrics and Gynecology, Women's Wellness and Research Centre, Hamad Medical Corporation, Doha Qatar. Email: fminisha@hamad.qa; Department of Research, Women's Wellness and Research Centre, Hamad Medical Corporation, Doha Qatar; Department of Pediatrics and Neonatology, Women's Wellness and Research Centre, Hamad Medical Corporation, Doha, Qatar; >Chief Executive Officer, Women's Wellness and Research Centre, Hamad Medical Corporation, Doha, Qatar

**Keywords:** Cesarean complications, antenatal corticosteroids, preterm birth, Surgical morbidity, State of Qatar, repeat cesarean section

## Abstract

**Background:**

Cesarean delivery (CD) is associated with increased maternal and neonatal morbidity compared to vaginal delivery, particularly in cases classified as emergency procedures or when there are multiple CDs. This retrospective cohort study aims to examine the incidence of maternal and neonatal complications in women with multiple CDs.

**Methods:**

This study used data from a national perinatal database obtained from a single tertiary maternity care hospital. Women who delivered a singleton live birth after 24 weeks of gestation by CD were stratified into five groups based on the number of CDs, with the last group having five or more CDs. The women were divided into those with five or more CDs (Group 5) versus those with fewer than five (Groups 1 to 4). The maternal outcomes included intra-operative surgical complications, blood loss, and intensive care unit (ICU) admission. The neonatal outcomes included preterm birth, neonatal ICU (NICU) admission, respiratory distress syndrome (RDS), and perinatal death.

**Results:**

Of the 6,316 women in the study, 2,608 (41.3%) had a primary CD. 30.3%, 17.5%, and 7.3% of the cohort had their second, third, and fourth CDs, respectively. Women undergoing the 5^th^ CD and above formed the remaining 3.5% (227). Women in Group 5 had the highest risk of suffering a surgical complication (3.1%, p = 0.015) and postpartum hemorrhage (7.5%, p = 0.010). 24% of babies in Group 5 were born preterm (p < 0.001). They also had a 3.5 times higher risk of having a surgical complication (RR = 3.5, 95% CI 1.6-7.6, p = 0.002), a 1.8 times higher risk of developing postpartum hemorrhage (RR = 1.8, 95% CI 1.1-2.9, p = 0.014), a 1.7 times higher risk of delivering between 32-37 weeks of gestation (RR = 1.7, 95% CI 1.3-2.2, p < 0.001), a higher risk of the baby getting admitted to NICU (RR = 1.3, 95% CI 1.0-1.6, p = 0.038), and developing RDS (RR = 1.5, 95% CI 1.2-2.0, p = 0.002) compared to Groups 1-4. The risks of neonatal outcomes such as NICU admission (RR 2.9, 95% CI 2.1-4.0) and RDS (RR 3.5, 95% CI 2.3-5.5) were much higher in elective CDs performed at term compared to preterm births (p < 0.001 for both).

**Conclusion:**

Maternal morbidity significantly increases with the increasing number of CD. The increased risk of RDS and NICU admissions in the neonate with multiple CDs reflects lower gestational age and birthweight in these groups—consideration of preoperative steroids for lung maturation in these women to reduce neonatal morbidity warrants further discussion.

## Introduction

Cesarean delivery (CD) accounts for nearly 1 in 5 live births worldwide and is expected to increase to almost 30% by 2030^1^. Many maternal complications are associated with CD, an open abdominal surgery, including intraoperative and postoperative hemorrhage, sepsis, injury to the bladder, ureter, and bowel, thromboembolism, anesthesia complications, increased hospital length of stay, and maternal mortality^2^. Globally, associated maternal morbidity and mortality have decreased over recent decades due to advances in surgical care, the availability of blood, and the adoption of safe surgical techniques^3^. This advancement partially explains the rising trends, as CDs are increasingly performed even when medically not indicated. Factors such as advanced maternal age, maternal request, reluctance to accept an ill-perceived risk to the foetus in a vaginal delivery, increased rates of labor induction, declining rates of operative vaginal births, and improved access to health insurance contribute to the significant rise in primary CDs worldwide^4^.

The increasing primary cesarean rate subsequently leads to more women undergoing repeat CDs, as a previous cesarean is one of the most common indications for an elective CD^5,6^. In 2022, the Middle East region had an average fertility rate of 2.56 births per woman (the highest being 3.7 in Yemen)^7^. Despite a drop in fertility rates, the CD rate in the Middle East is reported to be as high as 40.6%^8^. Studies from Qatar report a CD risk of 25-30%, increasing to 46% in mothers with gestational diabetes^8,9^. The higher incidence of CDs and the desire for larger families result in more women having multiple cesareans.

Multiple CDs are associated with a higher risk of prolonged surgery time, intraoperative adhesions, maternal visceral injuries, hemorrhage, and blood transfusions, as well as poorer neonatal outcomes such as preterm birth, low APGAR scores, and admission to intensive care^10,11,12^, although the absolute risks remain low. Complications increase with an increased number of CDs^13^. The timing of elective delivery in such cases is often debated as it needs to be balanced between maternal risk and neonatal morbidity due to prematurity. The current trend is to plan an early-term elective CD in these women to reduce maternal morbidity associated with an unplanned procedure, but this often compromises neonatal outcomes. This study aims to fill the gap due to the lack of evidence from the Middle Eastern region. The maternal and neonatal effects associated with an increasing number of CDs are examined using registry data, and women with five or more CDs are compared with those with fewer than five.

## Material And Methods

### Study Design and Setting

This population-based study included women delivering at the largest public tertiary maternity hospital in Qatar, with an annual average of nearly 18,000 deliveries from January to April 2018. Data were extracted from the PEARL Peristat registry (Perinatal Neonatal Outcomes Research Study in the Arabian Gulf), a population-based registry funded by the Qatar National Research Fund (Grant number: NPRP 6-238-3-059) and approved by the Hamad Medical Corporation Institutional Review Board (IRB 13064/13). The registry records routinely collected hospital data for parturient women and their offspring. Approval extends to all secondary data analyses from the PEARL registry, with a waiver of informed consent.

### Participants

This study included all women with a singleton pregnancy who delivered a live birth by a CD after 24 weeks of gestation (age of viability) from January 2017 to April 2018, with no other exclusion criteria. These women were then stratified into five groups based on the number of CDs, with the last group including women with ≥ 5 CDs (Groups 1 to 5). The women were also categorized into two groups: those with five or more CDs (Group 5) versus women with fewer than five CDs (Groups 1 to 4) as the baseline. This binary division was performed to study the risks of complications in women with five or more CDs, as this group is specifically less studied.

### Data Source and Variables

After applying the inclusion and exclusion criteria, all data were extracted from the PEARL-Peristat registry dataset. Independent data collectors recorded information from electronic patient health records into the registry. To maintain patient confidentiality, patient-identifying variables such as name, hospital number, and date of birth were not extracted for this study.

Demographic variables included age at delivery in years, parity (number of previous deliveries beyond the age of viability), nationality (categorized into Qataris and non-Qataris), gestational age (GA) at delivery in completed weeks, type of CD (elective CD, defined as a planned procedure, or emergency CD, defined as an unplanned CD performed due to a maternal or fetal complication), birthweight measured immediately after birth in grams, and antenatally diagnosed abnormal placentation (including placenta previa, defined as the placenta inserted partially or wholly in the lower uterine segment, and morbidly adherent placenta, defined as placenta that becomes abnormally adherent to the underlying uterine wall).

Maternal outcome variables were as follows:

• Surgical complications (SC): A composite outcome, any one of injury to the bladder or bowel, the need for hysterectomy, pelvic or abdominal wall hematoma, requirement for return to theatre for a repeat laparotomy, and postoperative sepsis (dichotomous variable yes/no). These complications were grouped into one composite outcome due to the very low prevalence of each complication.
• Estimated blood loss (EBL) (ml): Estimated by the operating theatre nursing staff, combining blood in the suction apparatus, gauze count, and any extra blood pooled in the patient's surroundings.
• Postpartum hemorrhage (PPH): EBL of more than 1000 ml (dichotomous, yes/no).
• Admission to the intensive care unit (ICU): requiring transfer for respiratory or cardiovascular support after the surgery (dichotomous, yes/no).

Fetal/neonatal outcomes included the following dichotomous variables (yes/no):

• Preterm birth (PTB)^14^:a) Late preterm birth: gestational age at birth between 32 to 37 completed weeks.b) Very preterm birth: gestational age at birth less than 32 completed weeks.
• Low birthweight (LBW): defined as birthweight < 2500 grams.
• APGAR score less than seven at 5 minutes of birth.
• Admission to the neonatal intensive care unit (NICU) for any reason.
• Respiratory distress syndrome (RDS): diagnosed and documented by the attending neonatologist.

### Statistical Analysis

All continuous variables (age, parity, gestational age at birth, birthweight, estimated blood loss) were reported as mean ±  standard deviation (SD) or median ±  interquartile range (IQR), depending on the distribution. The distributions were analyzed using histograms and the Shapiro-Wilk test.

Categorical variables were reported as frequency and percentage of the total women in each comparison group. Demographic data were compared between the five CD groups. Normally distributed data were compared using one-way ANOVA, while non-normally distributed data were compared using the Kruskal-Wallis test. Categorical variables were compared using the Chi-square test or Fisher's exact test (when the expected cell values were less than five) as appropriate.

Outcome variables were also reported and compared similarly between the five CD groups. Generalized linear models (GLM) for the binomial family were used to calculate risk ratios (RR) and 95% confidence intervals (CI) comparing the perinatal outcomes between women with five or more CDs and those with fewer than five. The RRs and 95% CIs were reported separately for elective and emergency CDs.

A p-value < 0.05 was considered strong evidence against the null hypothesis of no difference beyond chance (statistically significant). All statistical analyses were done using STATA statistical software, Release 16^15^.

## Results

A total of 6,316 women were included in the analysis. Of these, 2,608 (41.3%) women had a primary CD (Group 1). The cohort included 30.3%, 17.5%, and 7.3% who underwent their second, third, and fourth CD (Groups 2, 3, 4). Due to the small number, women with five or more CDs were grouped (Group 5), contributing only 3.6% (227) to the sample. Among them, 44, 108, and 2 women underwent the 6^th^, 7^th^, and 8^th^ CD, respectively.

The maternal demographics in each comparison group are presented in Table 1. Women in Group 5 were older, with a mean maternal age of 36 years compared to 30 years in Group 1, and had higher parity. Although primary CD rates were lower among the Qatari nationals, 55.5% of cases in Group 5 were Qatari nationals. Except for Group 1, CDs were more likely to be elective than emergency procedures in all other groups. With increasing cesareans, women had a higher chance of giving birth earlier (mean gestational age was 36.8 ± 1.9 completed weeks for Group 5 compared to 37.9 ± 2.7 weeks in Group 1). These women also had a significantly higher incidence of placenta previa (3.5% in Group 5 versus 1.7% in Group 1) and abnormal placentation (0.9% in Groups 4 and 5 versus 0.04% in Group 1). All these differences were statistically significant.

Maternal and fetal outcomes based on the number of CDs have been outlined in Table 2. In total, 61 women developed at least one surgical complication (adhesions, injury to the bladder, bowel or ureter, hysterectomy, pelvic hematoma, return to theatre, or sepsis)-an incidence of 9.6 per 1000 cesarean births. Women in Group 5 had the highest incidence of experiencing a surgical complication at 31 per 1,000 (p = 0.015). This group was also at a higher risk of bleeding, with 7.5% of women in Group 5 documented to have had a postpartum hemorrhage (p = 0.010). However, there was no statistically significant difference in the proportion of women admitted to the intensive care unit between the groups.

The higher the number of CDs a woman had, the more likely was the risk of a preterm birth. Nearly 24% of babies in Group 5 were born at less than 37 weeks (p < 0.001). Group 1 had the highest incidence of LBW (18.3%) and NICU admission (26.4%) compared to the other groups. The incidence of LBW then ranged from 9.9% in Group 2 to nearly 14% in Groups 4 and 5 (p < 0.001). The risk of NICU admissions ranged from 14% in Group 2 to 25.6% in Group 5 (p < 0.001). The highest incidence of neonatal RDS was seen in Group 5 (18.9%; p < 0.001). No statistically significant difference was noted in the proportion of babies with an APGAR score < 7 at 5 minutes.

**Figure 1.**
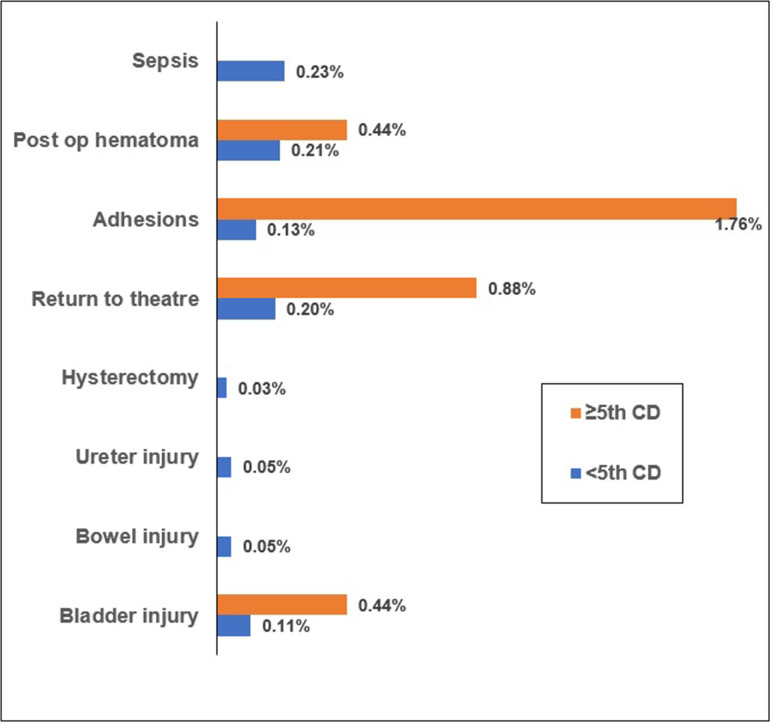
The proportion of women with surgical complications in the exposure groups; CD: Cesarean delivery.

The cesarean groups were dichotomized, and outcomes between Group 5 and the combined data from other groups (Groups 1-4) were compared. Women in Group 5 had a higher risk of composite surgical complications (3.1% vs 0.9%; p = 0.002), as shown in Table 3. The proportions of women with various surgical complications in each group are illustrated in Figure 1, indicating higher risks of intraoperative adhesions, postoperative pelvic hematomas, return to theatre for a re-laparotomy, and bladder injury in Group 5. Approximately 24% of women in this group gave birth prematurely (versus 15.3%; p < 0.001) and had a higher risk of babies with RDS (18.9% vs 12.3%; p = 0.002) and admission to NICU (25.6% vs 20.1%; p = 0.038).

**Figure 2.**
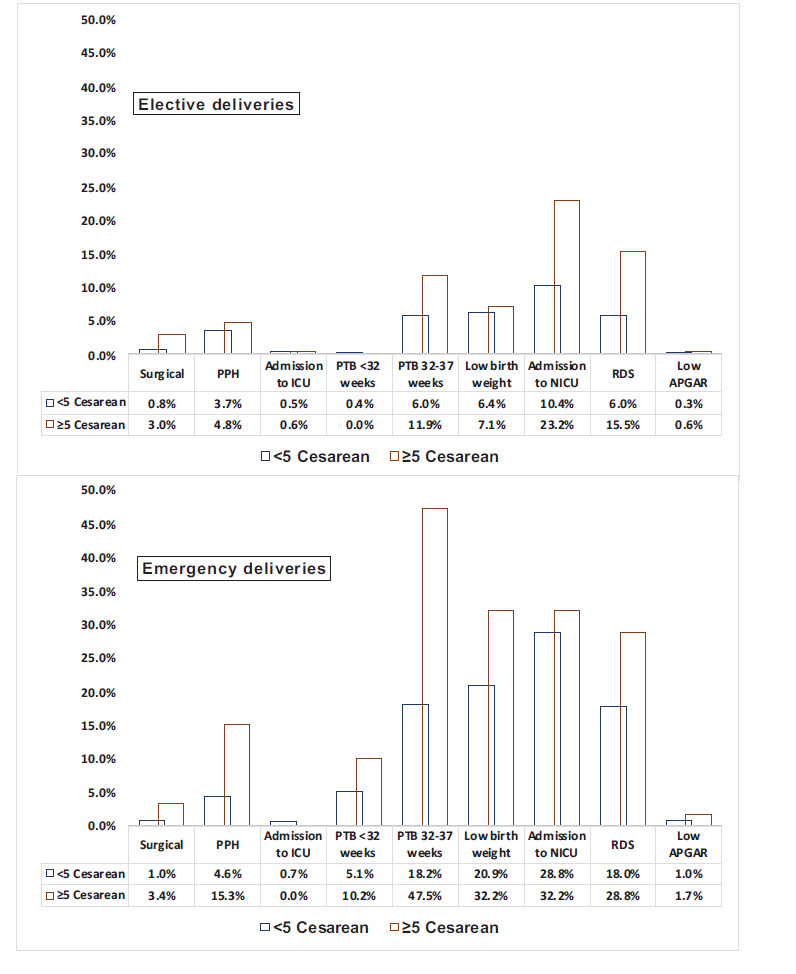
Comparison of percentages of maternal and neonatal groups between women with < 5 CD and ≥ 5 CD, undergoing elective and emergency deliveries; CD- cesarean delivery, PPH- postpartum hemorrhage; ICU- intensive care unit; PTB- preterm birth; NICU- neonatal intensive care unit, RDS- respiratory distress syndrome.

Outcomes were compared separately for elective and emergency deliveries, as depicted in Figure 2. Overall, the proportions of women with the results in each group were reduced with elective procedures compared to emergency delivery. In women having an emergency delivery, Group 5 had a 57.7% risk of PTB (vs. 11.9% in elective; p < 0.001), a 32.2% risk of LBW and NICU admission (vs. 7.1%, p < 0.001 and 23.2%, p = 0.173 in elective, respectively), a15.3% risk of PPH (vs. 4.8% in elective; p = 0.009) and 28.8% risk of RDS (vs. 15.5% in elective; p = 0.025). In Group 5, having an elective delivery, 23.2% of women had the baby admitted to NICU, with nearly 15.5% suffering from RDS (vs. 10.4% and 6.0% in Groups 1-4; p < 0.001 for both).

Results from the GLMs are presented in Table 3. Overall, Group 5 had a 3.5 times higher risk of having surgical complications (RR = 3.5, 95% CI 1.6-7.6, p = 0.002), a 1.8 times higher risk of developing PPH (RR = 1.8, 95% CI 1.1-2.9, p = 0.014), a 1.7 times higher risk of delivering between 32-37 weeks of gestation (RR = 1.7, 95% CI 1.3-2.2, p < 0.001), a higher risk of admission to NICU (RR = 1.3, 95% CI 1.0-1.6, p = 0.038), and developing RDS (RR = 1.5, 95% CI 1.2-2.0, p = 0.002).

In women undergoing an elective cesarean, the RRs of admission to NICU (RR = 2.2, 95% CI 1.7-3.0, p < 0.001) and developing RDS (RR = 2.6, 95% CI 1.8-3.8, p < 0.001) were higher than those undergoing emergency CD. Similarly, the RRs of developing PPH (RR = 3.4, 95% CI 1.8-6.2, p < 0.001), delivery between 32-37 weeks of gestation (RR = 2.6, 95% CI 2.0-3.4, p < 0.001), and low birth weight (RR = 1.5, 95% CI 1.1-2.2, p = 0.025) were higher in women undergoing emergency CD. The increased risk of surgical complications in Group 5 was similar in elective and emergency procedures.

Neonatal outcomes in elective CDs were analyzed separately for preterm and term deliveries, as shown in Table 4. In term births, babies born to women in Group 5 had a 2.9 times higher risk for NICU admission (RR 2.9, 95% CI 2.1-4.0, p < 0.001) and a 3.5 times higher risk for RDS (RR 3.5, 95% CI 2.3-5.5 p < 0.001). In preterm births, there were no statistically significant differences between groups in the neonatal outcomes.

## Discussion

The study results indicate that women with multiple CDs faced an increased risk of maternal morbidity, including surgical complications such as visceral injury, adhesions, pelvic hematomas, return to theatre, and postpartum hemorrhage. However, the absolute incidence remained small ( < 1%), with no increase in the risk of maternal admission to intensive care (Table 2). Babies born to these women had a higher risk of prematurity, admission to NICU, and respiratory distress (Table 3); term babies were at a significantly higher risk of NICU admission and RDS in elective CDs (Table 4).

Over the past decades, Qatar has witnessed a steady rise in cesarean rates, with reports showing nearly 1 in 3 live births resulting from a CD^16^. Despite a declining fertility rate^7^, having larger families remains a societal norm. The perception within healthcare that CD is a risk-free procedure contributes to the increasing trend of multiple CDs. This paper reports 227 women with five or more CDs, a higher number than previously documented in the literature. This study aims to quantify the increased risks associated with women undergoing more CDs to facilitate adequate patient counseling and guide reforms in clinical practice.

The findings reported here align with those of other studies in the Middle East. A retrospective case-control study conducted in Saudi Arabia in 2013 found an increased incidence of uterine rupture and intraoperative complications in women undergoing their 4th or more CD compared to those with 2-3 CDs. Unlike in this study, they did not find significant differences in placenta accreta and visceral injury risks. They also reported a higher risk of prematurity and low APGAR scores ( < 7 at 5 minutes) in the study group; however, there is no difference in the risk of NICU admission^10^. In a similar vein, Osman et al. conducted a comparison between 80 women who underwent their 6th or subsequent cesarean sections and 80 women who had their third to fifth cesarean deliveries between 2006 and 2010 in a tertiary care setting in Saudi Arabia. Their findings reported that outcomes for maternal and neonatal aspects were similar to the results observed in this paper^17^. Kaplanoglu et al. identified four or more CDs as the critical level for most maternal complications^18^. However, none of these studies reports the differences between elective and emergency procedures.

Other studies have also used the critical level of a 4th cesarean. A 2013 national UK study comparing 92 women undergoing their 4^th^ or more CD with those who had fewer CDs reported a similar risk of visceral injury, postpartum hemorrhage, and preterm delivery in the group with the highest number of CDs^11^. However, in contrast to the findings in this research, they demonstrate a 2.7 times higher risk of ICU admission. This difference could be explained by the variations in subjective ICU admission criteria in different settings and possibly due to advances in surgical care made over the years. Similarly, studies conducted in Pakistan and Egypt also show that maternal complications such as placenta previa, bladder injury, and dense intraabdominal adhesions were significantly higher in women undergoing their 4^th^ cesarean or higher^19,20^. The data in this study revealed that women having five or more CDs had a statistically significant rise in maternal morbidity. The American College of Obstetricians and Gynecologists (ACOG) committee opinion on CD due to maternal request recommends informing pregnant women requesting repeat cesarean delivery about the risks associated with higher order CDs such as placenta previa, accreta, and hysterectomy^21^.

One of the main findings in this paper is the higher risk of NICU admission and RDS in mothers who underwent multiple elective CDs, even after adjusting for preterm birth. Neonates born after elective cesarean have been shown to have a higher risk of respiratory morbidity and a requirement for critical care^22^. A systematic review analyzing more than 55,000 newborns delivered by CD reported a 95% increased risk of neonatal morbidity compared to babies delivered vaginally^23^. Moreover, evidence states that elective CD before 39 weeks is associated with higher neonatal morbidity, including NICU admission and RDS^24^. The authors recommend delaying elective births beyond 39 weeks of gestation. Despite this, in women with multiple previous cesareans, there is an inclination for clinicians to consider early-term deliveries as a way of reducing maternal morbidity due to the increased risk of uterine rupture and unplanned delivery associated with advanced gestational age in a scarred uterus. A 2022 study reported that elective delivery between 38-39 weeks is a good balance between the maternal morbidity associated with prolonging the pregnancy beyond 39 weeks and the neonatal morbidity associated with delivery before 38 weeks^25^.

Most women in this study sample with five or more CDs had early-term deliveries (mean GA of 37 weeks), which might explain the increased neonatal risks. The value of antenatal steroids in near-term elective cesareans is still considered questionable. The Royal Colleges of Obstetricians and Gynecologists (RCOG) Greentop guidance on antenatal corticosteroids for fetal lung maturity states that there is insufficient evidence of neonatal benefit from antenatal steroids before elective cesareans between 37 and 39 weeks of gestation^26^. However, this conclusion was based on a systematic review of a single small trial including 942 neonates born by elective cesarean. The results showed a 65% reduction in RDS but with low precision and wide CIs, most likely due to the small sample size^27^. These results were in contrast to previous reviews that showed a benefit beyond 37 weeks when given before elective CDs^28^. Prolonging the pregnancy until 39 weeks, as recommended^24^, in women with multiple CDs is impractical and associated with maternal morbidity^25^. Considering the results from this study showing an increased risk of RDS and NICU admission in term elective CDs in these women, the requirement for antenatal steroids in these cases needs to be considered.

### Strengths and Limitations

This study is the first from Qatar to evaluate perinatal outcomes in women with multiple CDs. The women included in this study are representative of the pregnant population of the country, as nearly 80% of the deliveries occur at the study site. The Peristat registry data was extracted meticulously by trained data collectors. Missing data, when present, was < 1% and, therefore, did not affect the interpretation of the outcomes. The large numbers give us enough power to detect meaningful differences in otherwise rare outcomes. The women were grouped based on their mode of birth, making it unlikely for any misclassification. The outcomes are morbidity events that must be documented in detail in patient files; hence, it's doubtful that any outcomes were missed or misclassified.

However, various studies have been conducted worldwide that address this research question^11,12,13,19,20,29^. The analysis in this paper quantifies the adverse outcomes in women having five or more CDs and differentiates between elective and emergency procedures, neither of which have been done before. Specific consequences like intraoperative adhesions, blood loss during surgery, return to theatre, etc., can be subjective based on the clinical situation and the medical staff's expertise in the management. However, this is unlikely to be a significant problem, as the hospital follows various pre-, intra, and postoperative protocols and checklists to ensure standardized clinical management. It is often challenging to compare certain clinical outcomes between studies from different settings due to differences in clinical protocols and healthcare systems. Additionally, this study used data retrospectively obtained from patient files as documented by healthcare professionals and is limited by the quality, consistency, and intra-practitioner variability of reporting in clinical records.

## Conclusion

Women with multiple cesareans face an elevated risk of maternal surgical complications such as adhesions, visceral injury, pelvic hematomas, return to theatre, sepsis, and PPH. This morbidity is notably higher in women undergoing five or more CDs, whether through elective or emergency deliveries. Furthermore, the higher the number of CDs, the earlier the GA is at delivery. Neonates born to these women also exhibit an increased risk of admission to NICU and RDS, even in planned elective deliveries at term. It is critical to inform women about the substantial morbidity associated with multiple CDs, emphasizing that while the absolute incidence is low, contraceptive advice should be offered. Given the tendency for planned early-term deliveries in this high-risk group, consideration should be given to providing antenatal corticosteroids for fetal lung maturity in this high-risk group to reduce neonatal morbidity.

### Acknowledgement

We would like to acknowledge the members of the PEARL Peristat registry group (Dr. Tawa Oluwade, Dr. Mai AlQubaisi, Dr. Sawsan Al Obaidly, Dr. Husam Salam, and the data collection team) for providing us with a high-quality data set for this study.

### Authors contributions

Authors TF, SAQ, NAD, and HAR conceptualized the study. SA, FM, and TF were involved in data accrual and processing. FM, TF, and NAD were involved in data analysis and presentation; SA and FM prepared the initial draft of the manuscript. Finally, all authors reviewed, edited, and approved the final version.

### Conflicts of interest

The authors have no conflicts of interest to declare.

## Figures and Tables

**Table 1:** Maternal demographics in the five Cesarean delivery groups (Group 1 to Group 5).

Demographics		Number of Cesarean deliveries										p-value
		Group 1: 1 N = 2,608		Group 2: 2 n = 1,912		Group 3: 3 N-1,108		Group 4: 4 N = 461		Group 5: ≥ 5 N = 227		
		n	%N	n	%N	n	%N	n	%N	n	%N	
Maternal age in years (Mean ± SD)		29.9 ± 5.7		31.2 ± 5.1		32.3 ± 4.6		33.8 ± 4.5		35.9 ± 4.1		< 0.001*
Parity (Mean ± SD)		1.1 ± 1.7		1.8 ± 1.5		2.5 ± 1.1		3.4 ± 0.9		4.7 ± 1.2		0.001*
Nationality	Qatari	818	31.4	549	28.7	352	31.8	181	39.3	126	55.5	< 0.001*
	Non-Qatari	1,789	68.6	1,362	71.3	755	68.2	280	60.7	101	44.5	
Type of Cesarean	Elective	599	23.0	1,157	60.5	807	72.8	316	68.6	168	74.0	< 0.001*
	Emergency	2,009	77.0	755	39.5	301	27.2	145	31.5	59	26.0	
Gestational age at birth (weeks) (Mean ± SD)		37.9 ± 2.7		38.1 ± 2.0		37.5 ± 1.8		37.1 ± 2.0		36.8 ± 1.9		< 0.001*
Birthweight grams (Mean ± SD)		3,044 ± 708		3,198 ± 586		3,139 ± 575		3,068 ± 595		3,021 ± 568		< 0.001*
Placenta praevia #		44	1.7	18	0.9	17	1.5	13	2.8	8	3.5	0.004*
Abnormal placentation #		1	0.04	9	0.5	6	0.5	4	0.9	2	0.9	0.001*

CD- Cesarean delivery; SD- standard deviation; #- compared using Fishers exact; all other categorical by Chi-square; All continuous compared using one-way ANOVA; p < 0.05 considered evidence against null hypothesis.

**Table 2:** Maternal and fetal outcomes in the five Cesarean delivery groups (Group 1 to Group 5).

Maternal and fetal outcomes		Number of Cesarean deliveries										p-value
		Group 1: 1 N = 2,608		Group 2: 2 n = 1,912		Group 3: 3 N-1,108		Group 4: 4 N = 461		Group 5: ≤ 5 N = 227		
		n	%N	n	%N	n	%N	n	%N	n	%N	
Surgical complications (any injury to the bladder, bowel, ureter, hysterectomy, hematoma, return to theatre, or sepsis)		20	0.8	17	0.9	15	1.4	2	0.4	7	3.1	0.015*
Estimated blood loss (Median ± IQR)		400 ± 250		350 ± 200		300 ± 100		350 ± 200		400 ± 200		< 0.001*
Postpartum hemorrhage		129	5.0	64	3.4	42	3.8	17	3.7	17	7.5	0.010*
Admission to intensive care #		21	0.8	5	0.3	8	0.7	1	0.2	1	0.4	0.106
Gestational age at delivery	Preterm < 32 weeks	108	4.1	31	1.6	21	1.9	16	3.5	6	2.6	< 0.001*
	Preterm 32-37 weeks	348	13.3	190	9.9	138	12.5	81	17.6	48	21.2	
	Term >37 weeks	2,152	82.5	1,691	88.4	949	85.7	364	79.0	173	76.2	
Low birth weight ( < 2500gms)		477	18.3	190	9.9	125	11.3	64	13.9	31	13.7	< 0.001*
NICU admission		688	26.4	265	13.9	174	15.7	96	20.8	58	25.6	< 0.001*
Respiratory distress		390	15.0	159	8.3	123	11.1	77	16.7	43	18.9	< 0.001*
APGAR 5 min less than 7 #		23	0.9	10	0.5	4	0.4	3	0.7	2	0.9	0.344

CD- Index Cesarean delivery; IQR- interquartile range; NICU- neonatal intensive care unit #- compared using Fishers exact; all other categorical by Chi square; Estimated blood loss compared using Kruskal Wallis test; p < 0.05 considered evidence against the null hypothesis.

**Table 3:** Crude Risk Ratios comparing ≥ 5 Cesarean (Group 5) with < 5 Cesarean (Groups 1-4), both elective and emergency.

Maternal-fetal complications	Group 1-4 N=6,089 (Baseline)		Group 5 N=227		Overall N=6,316		Elective Cesarean N=3,047		Emergency Cesarean N=3,269	
	n	%N	n	%N	RR (95% CI)	p-value	RR (95% CI)	p-value	RR (95% CI)	p-value
**Surgical complications (any injury to the bladder, bowel, ureter, hysterectomy, hematoma, return to theatre, or sepsis)**	54	0.9	7	3.1	**3.5 (1.6-7.6)**	0.002*	3.9 (1.5-10.2)	0.005*	3.4 (0.8-13.9)	0.088
**Postpartum hemorrhage**	252	4.1	17	7.5	**1.8 (1.1-2.9)**	0.014*	1.3 (0.6-2.6)	0.472	**3.4 (1.8-6.2)**	< 0.001*
**Admission to intensive care #**	35	0.6	1	0.4	0.8 (0.1-5.6)	0.793	-	-	-	-
**Preterm birth < 32 weeks #**	176	2.9	6	2.6	0.9 (0.4-2.0)	0.827	-	-	-	-
**Preterm birth 32-37 weeks**	757	12.4	48	21.2	**1.7 (1.3-2.2)**	< 0.001*	2.0 (1.3-3.0)	0.002*	**2.6 (2.0-3.4)**	< 0.001*
**Low birth weight ( < 2500gms)**	856	14.1	31	13.7	1.0 (0.7-1.4)	0.864	1.1 (0.6-2.0)	0.699	**1.5 (1.1-2.2)**	0.025*
**NICU admission**	1,223	20.1	58	25.6	**1.3 (1.0-1.6)**	0.038*	**2.2 (1.7-3.0)**	< 0.001*	1.1 (0.8-1.6)	0.557
**Respiratory distress**	749	12.3	43	18.9	**1.5 (1.2-2.0)**	0.002*	**2.6 (1.8-3.8)**	< 0.001*	1.6 (1.1-2.4)	0.023*
**APGAR 5 min less than 7**	40	0.7	2	0.9	1.3 (0.3-5.5)	0.684	-	-	-	-

Baseline: Baseline groups in the calculation of risk ratios; CD: Cesarean delivery; NICU: Neonatal intensive care unit; RR: Risk Ratios; CI: confidence interval; #: 0 cases in one strata; p < 0.05 is evidence against null hypothesis of no difference.

**Table 4:** Risk ratios for neonatal outcomes in elective cesarean deliveries.

Neonatal outcomes in elective deliveriesGroup 5 versus Groups 1-4		Risk Ratios (95% CIs)	p-value
**Low birth weight ( < 2500gms)**	Preterm	0.4 (0.2-1.0)	0.043
	Term	1.5 (0.8-3.1)	0.233
**Neonatal intensive care unit admission**	Preterm	0.7 (0.4-1.2)	0.202
	**Term**	**2.9 (2.1-4.0)**	** < 0.001**
**Neonatal respiratory distress syndrome**	Preterm	0.8 (0.4-1.6)	0.493
	**Term**	3.5 (2.3-5.5)	** < 0.001**

CI- confidence intervals; Preterm (Delivery < 37 weeks gestation) N = 210; Term N = 2843; p < 0.05 provides evidence against the null hypothesis.

**Table:** Table of abbreviations

ANOVA	Analysis of variance
APGAR	Appearance, pulse, grimace, activity, respiration
CD	Cesarean delivery
CI	Confidence intervals
EBL	Estimated blood loss
GA	Gestational age
GLM	Generalized linear models
ICU	Intensive care unit
IQR	Interquartile range
LBW	Low birth weight
NICU	Neonatal intensive care unit
PEARL	Perinatal Neonatal Outcomes Research Study in the Arabian Gulf
PPH	Postpartum hemorrhage
RDS	Respiratory distress syndrome
RR	Risk ratios
SC	Surgical complications
SD	Standard deviation
